# Pharmacokinetic Analysis of ^64^Cu-ATSM Dynamic PET in Human Xenograft Tumors in Mice

**DOI:** 10.3390/diagnostics5020096

**Published:** 2015-03-27

**Authors:** Fan Li, Jesper Tranekjær Jørgensen, Jacob Madsen, Andreas Kjaer

**Affiliations:** 1 Cluster for Molecular Imaging, Faculty of Health Sciences, University of Copenhagen, Blegdamsvej 3, 2200 Copenhagen, Denmark; E-Mails: jtran@sund.ku.dk (J.T.J.); akjaer@sund.ku.dk (A.K.); 2 Department of Clinical Physiology, Nuclear Medicine & PET, Rigshospitalet, University of Copenhagen, Blegdamsvej 9, 2100 Copenhagen, Denmark; E-Mail: Jacob.Madsen@regionh.dk (J.M.)

**Keywords:** kinetic modeling, ^64^Cu-ATSM, hypoxia, cancer, PET, PET/CT, xenograft tumors, voxel-wise pharmacokinetic analysis, parametric mapping

## Abstract

The aim of this study was to evaluate the feasibility to perform voxel-wise kinetic modeling on datasets obtained from tumor-bearing mice that underwent dynamic PET scans with ^64^Cu-ATSM and extract useful physiological parameters. Methods: Tumor-bearing mice underwent 90-min dynamic PET scans with ^64^Cu-ATSM and CT scans with contrast. Irreversible and reversible two-tissue compartment models were fitted to time activity curves (TACs) obtained from whole tumor volumes and compared using the Akaike information criterion (AIC). Based on voxel-wise pharmacokinetic analysis, parametric maps of model rate constants *k*_1_, *k*_3_ and *K*_i_ were generated and compared to ^64^Cu-ATSM uptake. Results: Based on the AIC, an irreversible two-tissue compartment model was selected for voxel-wise pharmacokinetic analysis. Of the extracted parameters, *k*_1_ (~perfusion) showed a strong correlation with early tracer uptake (mean spearman *R* = 0.88) 5 min post injection (pi). Moreover, positive relationships were found between late tracer uptake (90 min pi) and both *k*_3_ and the net influx rate constant, *K*_i_ (mean spearman *R* = 0.56 and *R* = 0.86; respectively). Conclusion: This study shows the feasibility to extract relevant parameters from voxel-wise pharmacokinetic analysis to be used for preclinical validation of ^64^Cu-ATSM as a hypoxia-specific PET tracer.

## 1. Introduction

Tumor hypoxia is a key factor in the development of aggressive and therapy-resistant tumors [1,2,3]. Several techniques have therefore been applied for the evaluation of intratumoral oxygen tension with the purpose of improving treatment responsiveness, and for possible use in individualized treatment planning. Invasive oxygen electrode measurements provide direct quantitative information of tumor oxygenation and are generally considered the gold standard for the detection of tumor hypoxia [4,5,6,7]. However, this method is technically demanding and depends on the accessibility of the tumor for probe insertions [8]. Thus, there has been a search for alternative methods to assess tumor hypoxia.

PET imaging of the intratumoral microenvironment can be used for non-invasive tumor characterization and evaluation. At present, the majority of PET tracers used for imaging of hypoxia belongs to a group of compounds termed nitroimidazoles that are reduced and become trapped in hypoxic tissue [9,10,11]. [^18^F]Fluoromisonidazole (^18^F-FMISO) was the first nitroimidazole-based PET tracer to be developed and, therefore, also the most widely studied [10,12,13,14]. However, slow blood clearance means that this tracer produces rather low tumor-to-background ratios; a problem that has only partly been solved with different second generation nitroimidazoles that have been developed and evaluated [15,16,17,18].

**Figure 1:**
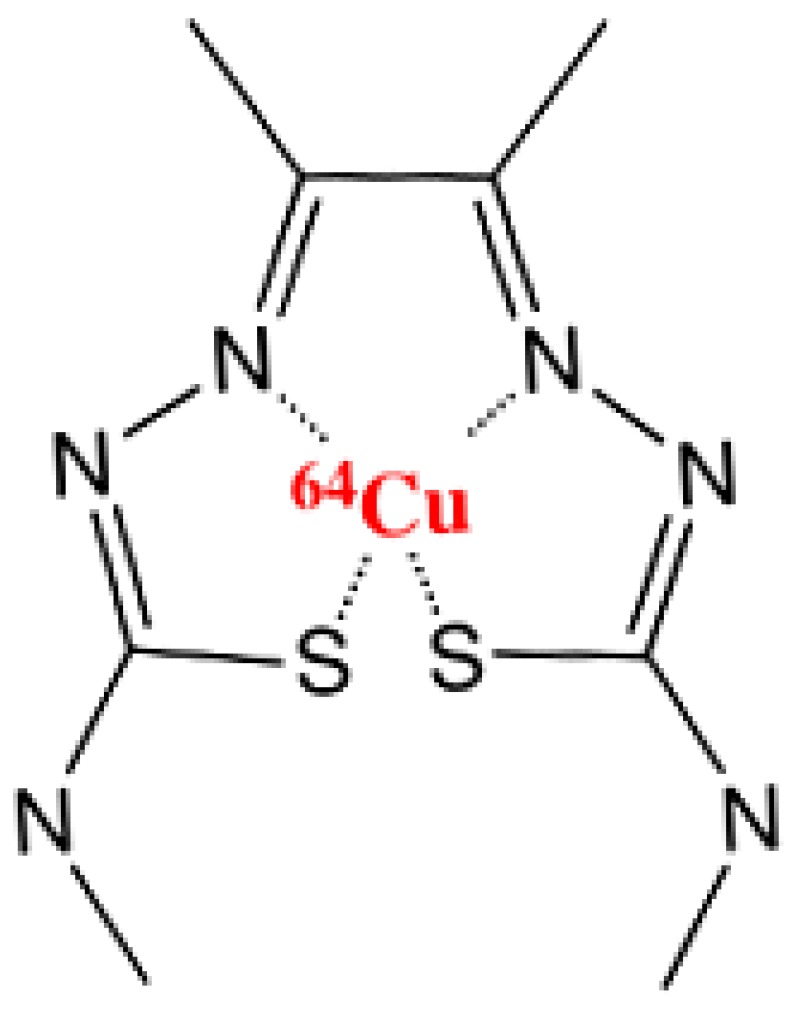
Chemical structure of hypoxia PET tracer ^64^Cu-ATSM.

Copper(II)-diacetyl-*bis*(*N*^4^-methylthiosemi-carbazone) (Cu-ATSM) (Figure 1), one of the few non-nitroimidazole-based compounds used for hypoxia PET imaging, has a high cellular permeability and rapid washout [19,20,21,22,23]. This tracer has shown promising results in a few clinical studies, where it has been used as a prognostic marker of treatment response in patients [24,25,26,27]. However, Cu-ATSM has also been evaluated preclinically in several cancer models, and these studies have reported variable results with regard to tissue type-dependent selectivity and temporal changes in tumor uptake [28,29,30]. The uptake mechanism is not fully understood, but it is believed that Cu(II)-ATSM enters cells by diffusion and is reduced to [Cu(I)-ATSM]^−^. Under normoxic condition, [Cu(I)-ATSM]^−^ is rapidly re-oxidized to Cu(II)-ATSM, and the compound is again able to leave the cell by diffusion. When there is a lack of oxygen, the tracer is not rapidly re-oxidized, and the unstable [Cu(I)-ATSM]^−^ can slowly dissociate and become trapped within hypoxic cells [31,32,33].

Visual assessment and semiquantitative approaches are routinely used for the analysis of static PET images, but do not account for variations in tracer concentration over time. However, this is relevant in regard to hypoxia PET tracers, as limited perfusion to hypoxic tumor areas can result in insufficient tracer delivery. Mathematic compartment modeling applied on dynamic PET data can be used to study tracer pharmacokinetics and extract information describing underlying physiological processes, such as tracer delivery, trapping and clearance [34,35]. In a few studies, kinetic modeling has been applied on PET data from nitroimidazole-based tracers to extract parameters that potentially can be used as surrogate markers of tumor hypoxia [35,36,37,38]. Even though this approach has shown promise, only a few studies have evaluated the model output against Po_2_ measurements or other imaging modalities [39,40]. Preclinical studies hold some advantages with regard to the availability of animal models of disease and the flexibility to apply multiple procedures before, during and after dynamic PET imaging. Therefore, despite some physical limitations due to the size, the ability to perform reliable kinetic modeling in mice would be attractive for the evaluation of model output.

In the present work, the feasibility to perform voxel-wise pharmacokinetic analysis on ^64^Cu-ATSM dynamic PET data from two human tumor xenograft models in mice was evaluated. Reversible and irreversible two-tissue compartment models were fitted to the PET data and the performance compared. Moreover, voxel-by-voxel nonparametric correlation analysis was used to compare the intratumoral spatial tracer distribution to relevant model parameters.

## 2. Methods and Materials

### 2.1. Animal Models

All animal experiments were approved by the Danish Animal Welfare Council, Ministry of Justice. Upon arrival, Naval Medical Research Institute (NMRI) nude mice (Taconic Europe, Lille Skensved, Denmark) were acclimatized for one week in the animal facility and had at all times access to water and chow *ad libitum*. Human colorectal cancer cells, HT29 (*n* = 2), (American Type Culture Collection, Manassas, VA, USA), and human neuroendocrine lung cancer cells, H727 (*n* = 2) (The European Collection of Cell Cultures, Salisbury, UK) were cultured at 37 °C and 5% CO_2_ in McCoy’s 5A and RPMI-1640 Glutamax medium with 10% fetal calf serum and 1% penicillin-streptomycin, respectively (all Invitrogen Ltd., Paisley, UK). Mice were inoculated with approximately 10^6^ of either HT29 or H727 cells, suspended in 200 μL (1:1 cell culture medium and BD™ Matrigel™ (VWR, Søborg, Denmark)), into each flank. Tumors were allowed to grow 2–3 weeks.

### 2.2. ^64^Cu-ATSM Administration and PET/CT Acquisition

^64^Cu was produced at Risø National Laboratory, Technical University of Denmark (Roskilde, Denmark). ^64^Cu-ATSM was synthesized at the Department of Clinical Physiology, Nuclear Medicine & PET, Rigshospitalet, Center of Diagnostic Investigations, Copenhagen, Denmark, as previously described [41]. Briefly, 1 mL of ^64^CuCl_2_ solution (in 0.1 M HCL) was mixed with 2 mL of 200 mM glycine buffer and was allowed to react for 4 min. Twenty microliters of H_2_-ATSM in dimethyl sulfoxide solution (1 mg/mL) were added to this mixture after the ^64^CuCl_2_-mixture was left to react at room temperature for 4 min. Finally, 5 mL of water were added, and the resulting mixture was loaded onto a SepPak light cartridge (Waters, Saint-Quentin, France). The cartridge was washed with 10 mL of water and was eluted with 1.2 mL 50% ethanol. The resulting product obtained a radiochemical yield >98% and was diluted in saline before administration in mice.

Animals were weighed, anaesthetized using a mixture of 3% sevoflurane (Abbott Scandinavia AB, Solna, Sweden) mixed with 35% O_2_ in N_2_ by breathing through a nosecone and placed on a bed platform. Their body temperature was kept stable by a heating pad. In order to ease image co-registration, three fiducial markers were placed around the scan bed. A vein catheter (26GA BD NeoFlon™, Becton Dickinson A/S, Albertslund, Denmark) was inserted into the tail vein, before the animals were placed in the center field of view of a small animal MicroPET Focus 120 scanner (Siemens Medical Solutions, Malvern, PA, USA). A 90-min PET acquisition was started, and ^64^Cu-ATSM was administrated as a bolus (17.21 ± 3.09 (mean ± SD) MBq) and the vein catheter removed. After the PET scan, mice had 0.2 mL of intravascular contrast agent (Fenestra™ VC^®^, ART advanced Research Technologies Inc., Saint-Laurent, Canada), administrated into a tail vein, and after a few minutes, the bed was moved to a small animal MicroCAT^®^ II system (Siemens Medical Solutions). A seven-minute CT scan was performed (X-ray voltage: 60 kVp; anode current: 500 μA; exposure time: 310 ms).

List-mode data were sampled into two sets of timeframe sequence definitions to finely sample for input function (15 × 2 s; 10 × 10 s; 5 × 80 s; 3 × 240 s; 3 × 960 s; 1 × 920 s) and tracer uptake in tumor regions (1 × 10 s; 1 × 20 s; 1 × 30 s; 4 × 60 s; 4 × 150 s; 15 × 300 s). 3D maximum *a posteriori* (MAP) based on 2D histogramming projectors was chosen as the method of reconstruction. 

### 2.3. Pharmacokinetic Analysis

PET and CT images were co-registered, and regions of interest (ROIs) were drawn using Inveon Research Workplace software (Siemens Medical Solutions). Guided by contrast-enhanced CT images, 20-pixel-sized circular regions of interest were placed within the left heart ventricle. Based on these, a volume of interest (VOI) covering the center of the cavity was created and used to generate the image-derived input function, C_p_.

For pharmacokinetic analysis, the dynamic PET data were imported into the PMOD 3.3 software (PMOD Technologies, Zurich, Switzerland). The input function was imported as a text file. For image fusion, an automatic predefined mouse-mode co-registration was applied, followed by visual inspection and, if necessary, manual fine-tuning. VOIs covering the target tissue were created by manually drawing, and the time activity curves (TACs) obtained from VOIs were subsequently imported to the PKIN package (PMOD) for the performance of kinetic modeling and kinetic parameter estimation. Nonlinear least squares method with the Levenberg-Marquardt optimization algorithm, which minimizes the weighted sum of squared errors between the obtained TACs and the predefined model, was used.

Irreversible and reversible 2-compartment kinetic models were applied to TACs obtained from VOIs covering whole tumor tissue, and the AIC was used to evaluate the models [42,43]. PKIN calculates AIC values and applies a correction for a small sample size. 

The PXMOD package (PMOD) was used for voxel-by-voxel analysis of the dynamic PET data. During import, the CT images were downscaled to match the pixel size of the PET images, and after image co-registration, the voxel size of all images were down-scaled further to 1.2 mm × 1.2 mm × 0.8 mm. Due to the large amount of information in the dynamic PET data, a new image, only including voxels within a cubic VOI placed around the tumor, was created. Irreversible 2-tissue compartment modeling with ridge-regression fitting was applied to the re-sized images to generate parametric maps of *k*_1_, *k*_3_, *K_i_*. The net influx, *K*_i_, is defined as *k*_1_ × *k*_3_/(*k*_2_ + *k*_3_). The average voxel uptake in corresponding tumor regions was collected at 0–5 min, 25–30 min and 85–90 min pi and compared to the values obtained from the parametric maps. 

### 2.4. Statistical Analysis

GraphPad Prism 5.03 (GraphPad Software, La Jolla, CA, USA) was used for statistical analysis. The intratumoral distribution of ^64^Cu-ATSM at different time points compared to kinetic parameters was assessed voxel-by-voxel using Spearman’s rank correlation analysis. Fishers weighted mean correlation coefficients were calculated. The significance level was set to 0.05, and Bonferroni correction for multiple testing was applied.

## 3. Results

### 3.1. ^64^Cu-ATSM Uptake

**Figure 2:**
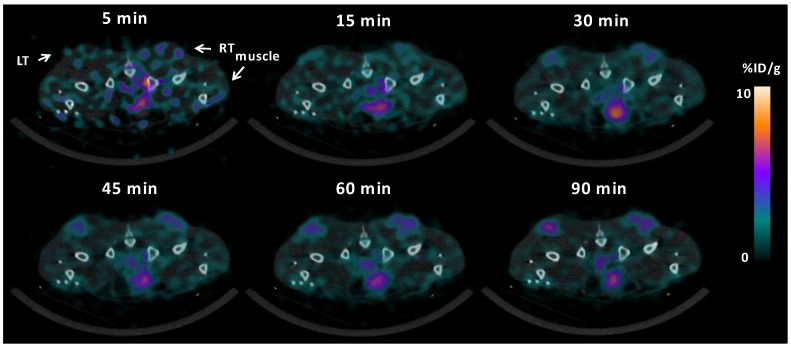
Dynamic PET image series with axial view of mice bearing subcutaneous neuroendocrine H727 tumors. White arrows point towards muscle and tumor areas (LT: left tumor and RT: right tumor).

In order to quantify ^64^Cu-ATSM uptake, VOIs covering tumor and muscle tissue were generated. The heterogeneous uptake pattern of ^64^Cu-ATSM was observed within intratumoral regions in all mice. However, while the uptake in muscle tissue remained relatively stable, the tumor uptake continued to increase over time, reaching an average tumor-to-muscle (T/M) ratio of 2.16 ± 0.74 (mean ± SD) at 90 min pi (Table 1 and Figure 2). Different trends were observed in the temporal increase of T/M-ratios between HT29 and H727 tumor-bearing mice. Generally, lower values were found in the mice with HT29 tumors at all time points. In the mice carrying HT29 tumors, an initial increase in the T/M ratios was seen between 15 min (0.86 ± 0.09) and 45 min (1.50 ± 0.21). However, the T/M ratio seemed to have reached a plateau after 45 min, and a similar level was found 90 min pi (1.62 ± 0.19). Contrarily, in the mice with H727 tumors, a continuous increase in T/M-ratios was seen for the duration of the scans, resulting in an approximately 2–3-fold higher uptake in tumor than muscle tissue 90 min pi (15 min: 1.31 ± 0.29; 45 min: 2.20 ± 0.59; 90 min: 2.71 ± 0.68).

### 3.2. Kinetic Analysis 

**Figure 3:**
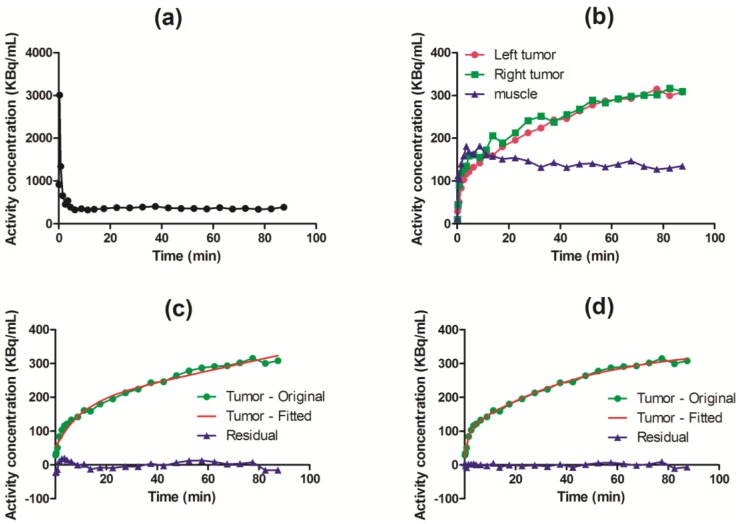
Time series data obtained from H727 xenografts tumor-bearing mice showing examples of: (**a**) TAC from the left ventricle cavity; (**b**) TACs from tumor (green and red) and muscle (blue) tissue; (**c**) tumor TAC (green) with corresponding fitting curve (red) to a two-tissue irreversible model and residuals (blue); (**d**) tumor TAC (green) with corresponding fitting curve (red) to a two-tissue reversible model and residuals (blue) from the analysis of a representative animal.

**Figure 4:**
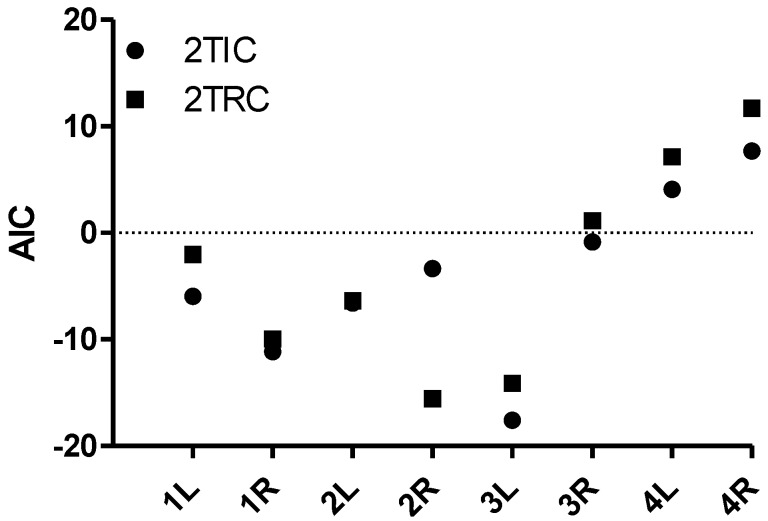
AIC calculated for the two-tissue irreversible compartment model (2TIC) and two-tissue reversible compartment model (2TRC) to TACs covering the whole tumor tissues from the PET studies. Lower AIC indicates better fit results of the model to the TAC data applied. PET scans were recorded dynamically 90 min in four mice, each bearing two subcutaneous tumors (number of the mouse corresponding to Table 1; followed by left (L) or right (R) tumor).

TACs were successfully generated by manual drawing ROIs, both covering the cavity of the left ventricle and the tumor regions. The image-derived input function was obtained for each animal. An example of a TAC derived from the left ventricle of a mouse is shown in Figure 3a. Figure 3b illustrates TACs obtained from target tissues based on the 90-min dynamic PET images. Two-tissue irreversible and reversible compartment models were fitted to TACs from tumor regions (Figure 3c,d), and AIC values were calculated, in order to compare the models. AIC takes into account the structural complexity of the model and goodness of fit. The model with the lowest calculated AIC value is considered to have achieved the optimal balance. Generally, the AIC values for the irreversible model were lowest (Figure 4), and based on this, it was chosen for the voxel-wise analysis. The estimated values for *k*_1_, *k*_2_, *k*_3_ and *K_i_* from the irreversible model on whole tumor tissue are shown in Table 1. The influx rate, *K*_i_, was the most stable among the kinetic parameters when all tumors were considered. This observation was also found within each tumor type.

**Table 1:** ^64^Cu-ATSM tumor-to-muscle (T/M) ratios and kinetic parameters *k*_1_, *k*_2_, *k*_3_ and *K*_i_ derived from 2-tissue irreversible compartmental modeling. White rows after animal number: left tumor; grey rows: right tumor.

Mouse		Tumor to muscle ratio					Irreversible model			
		15 min	30 min	45 min	60 min	90 min	*k*_1_	*k*_2_	*k*_3_	*K*_i_
							mL/cm^3^/min	1/min	1/min	mL/cm^3^/min
HT29	1	0.96	1.51	1.8	1.85	1.82	0.0613	0.0685	0.0027	0.0023
		0.91	1.15	1.34	1.41	1.37	0.0598	0.0892	0.0030	0.0020
	2	0.78	1.16	1.4	1.51	1.66	0.0547	0.0899	0.0065	0.0037
		0.78	1.05	1.45	1.39	1.63	0.0340	0.0535	0.0048	0.0028
H727	3	1.53	2.18	2.77	2.99	3.36	0.0532	0.0772	0.0063	0.0040
		1.57	2.11	2.61	2.7	3.21	0.0925	0.1643	0.0066	0.0036
	4	1.01	1.45	1.86	2.16	2.28	0.0375	0.0599	0.0071	0.0040
		1.11	1.37	1.55	1.76	1.97	0.0512	0.0903	0.0067	0.0035
All tumors	Mean	1.08	1.50	1.85	1.97	2.16	0.0555	0.0866	0.0054	0.0032
	SD	0.31	0.43	0.55	0.60	0.74	0.0178	0.0344	0.0017	0.0008
HT29 tumors	Mean	0.86	1.22	1.50	1.54	1.62	0.0524	0.0753	0.0042	0.0027
	SD	0.09	0.20	0.21	0.21	0.19	0.0126	0.0176	0.0017	0.0007
H727 tumors	Mean	1.31	1.78	2.20	2.40	2.71	0.0586	0.0979	0.0067	0.0038
	SD	0.29	0.43	0.59	0.55	0.68	0.0236	0.0460	0.0003	0.0003

### 3.3. Parametric Mapping and Voxel-by-Voxel Analysis

**Figure 5:**
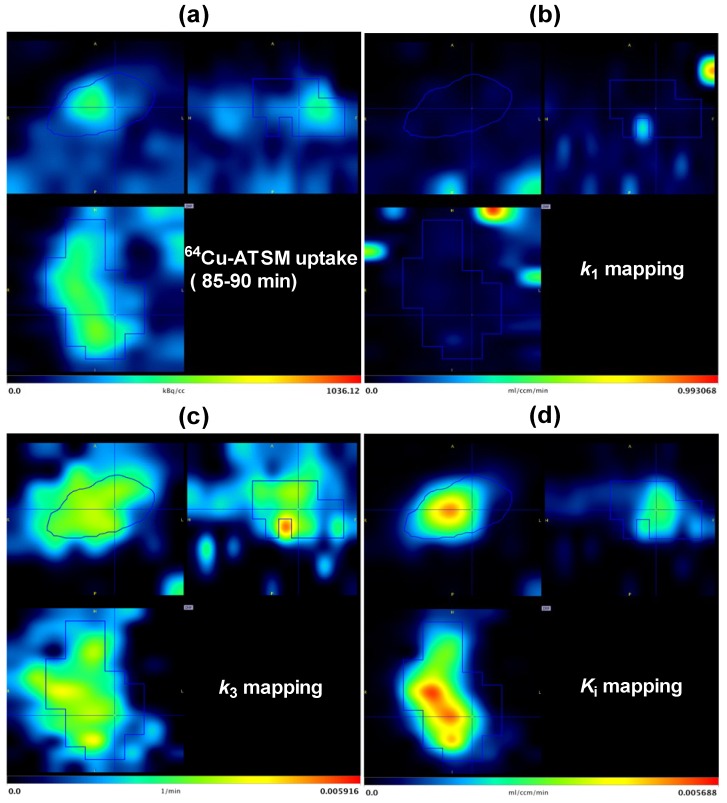
Matching images of ^64^Cu-ATSM uptake and parametric maps taken from Mouse 3 left tumor. Top left: transverse; Top right: sagittal; bottom left: coronal images. (**a**) ^64^Cu-ATSM uptake in tumor area at the last time frame (85–90 min); the corresponding parametric images of *k*_1_ (**b**), *k*_3_ (**c**) and net flux *K*_i_ (**d**).

The irreversible two-tissue compartment model was applied for voxel-wise analysis and parametric images of *k_1_*, *k_3_* and *K_i_* (Figure 5). Parametric images of *k_1_* demonstrated low values in intratumoral regions, but images also contained regions outside the tumor with a high value. It is possible that these could represent adjacent microvessels. As opposed to parametric maps of *k_1_*, generally, *k_3_* images showed relatively high values within tumor regions, including a few sub-volumes with very high intensity. However, there were also regions with high values outside the tumor, as well. In contrast, *K_i_* parametric images generally showed high tumor-to-background contrast with good delineation to the surroundings tissues.

**Figure 6:**
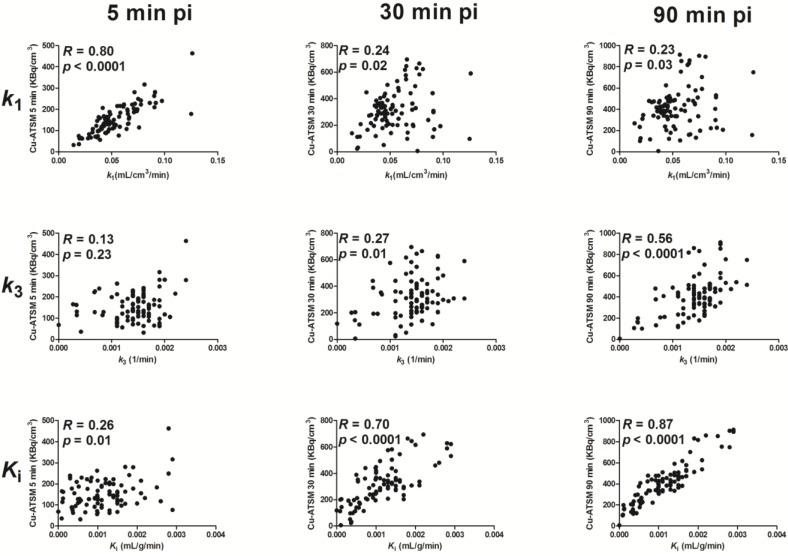
Example of voxel-by-voxel correlation analysis of ^64^Cu-ATSM uptake (kBq/cm^3^) (Mouse 3 left tumor). Top row: *k*_1_ (mL/cm^3^/min); middle row: *k*_3_ (min^−1^); bottom row: *K_i_* (mL/ cm^3^/min); left column: ^64^Cu-ATSM uptake 5 min pi; middle column: ^64^Cu-ATSM uptake 30 min pi; right column: ^64^Cu-ATSM uptake 90 min pi.

Voxel values were extracted from parametric maps and compared to PET uptake 0–5 min, 25–30 min and 85–90 min pi (Figure 6 and Table 2). The analysis showed a strong significant correlation between early ^64^Cu-ATSM uptake and *k*_1_ (5 min pi; 0.88 (mean); 0.80–0.94 (range)), but the relationship was not found after 30 and 90 min. In contrast, *k*_3_ showed moderate to strong correlations with ^64^Cu-ATSM uptake 90 min pi (0.59 (mean); −0.10–0.70 (range)), but not with early uptake. Finally, in some tumors, strong correlations were observed between *K*_i_ and ^64^Cu-ATSM uptake 30 min pi: (0.56 (mean); 0–0.71 (range)). This relationship was found in all tumors 90 min pi: (0.80 (mean); 0.41–0.89 (range)).

**Table 2:** Voxel-by-voxel comparison of the intratumoral spatial distribution obtained at 5, 30 and 90 min pi from dynamic ^64^Cu-ATSM scans and voxel intensity based on the kinetic parameter derived from parametric mapping. Correlation coefficients are presented with the confidence interval in brackets, and mean correlation coefficients are calculated as Fishers weighted mean correlation coefficients. All significant correlation coefficients are shown in bold (*p* < 0.002). HT29: *n* = 2; H727: *n* = 2.

Mouse			5 min *vs.* *k*_1_	30 min *vs.* *k*_1_	90 min *vs.* *k*_1_	5 min *vs.* *k*_3_	30 min *vs.* *k*_3_	90 min *vs.* *k*_3_	5 min *vs.* *K*_i_	30 min *vs.* *K*_i_	90 min *vs.* *K*_i_
**HT29**	**1**	**LT**	**0.91** (0.88–0.94)	**0.66** (0.54–0.75)	**0.55** (0.41–0.67)	**0.36** (0.19–0.51)	**0.52** (0.37–0.64)	**0.70** (0.56–0.77)	**0.46** (0.30–0.60)	**0.69** (0.57–0.78)	**0.82** (0.75–0.87)
		**RT**	**0.89** (0.84–0.93)	**0.41** (0.22–0.57)	0.25 (0.04–0.44)	0.31 (0.10–0.48)	**0.46** (0.27–0.61)	**0.65** (0.50–0.75)	**0.32** (0.12–0.49)	**0.59** (0.44–0.71)	**0.75** (0.64–0.83)
	**2**	**LT**	**0.88** (0.80–0.93)	−0.38 (−0.60–−0.10)	**−0.61** (−0.76–−0.40)	−0.14 (−0.34–−0.22)	−0.06 (−0.34–0.22)	**0.49** (0.24–0.68)	−0.18 (−0.44–0.11)	0 (−0.28–0.28)	**0.59** (0.37–0.74)
		**RT**	**0.94** (0.91–0.96)	**0.55** (0.39–0.68)	0.27 (0.07–0.45)	−0.16 (−0.35–0.05)	0.23 (0.03–0.41)	**0.58** (0.42–0.70)	0.04 (−0.17–0.24)	0.47 (−0.29–0.61)	**0.80** (0.71–0.86)
**H727**	**3**	**LT**	**0.80** (0.70–0.86)	0.24 (0.03–0.43)	0.23 (0.02–0.42)	0.13 (−0.09–0.33)	0.27 (0.06–0.46)	**0.56** (0.39–0.69)	0.26 (0.05–0.45)	**0.70** (0.57–0.79)	**0.87** (0.81–0.91)
		**RT**	**0.82** (0.76–0.87)	−0.08 (−0.24–0.08)	**−0.30** (−0.44–−0.14)	−0.28 (−0.43–−0.13)	**0.50** (0.36–0.61)	**0.73** (0.64–0.80)	−0.17 (−0.32–−0.01)	**0.71** (0.62–0.78)	**0.89** (0.85–0.92)
	**4**	**LT**	**0.83** (0.74–0.89)	**0.41** (0.20–0.59)	**0.47** (0.27–0.63)	−0.15 (−0.37–0.08)	**−0.45** (−0.62–−0.25)	−0.10 (−0.32–0.13)	0.17 (−0.07–0.38)	0 (−0.23–0.23)	**0.41** (0.20–0.58)
		**RT**	**0.90** (0.82–0.95)	0.21 (−0.10–0.49)	0.34 (0.04–0.59)	0.29 (−0.01–0.55)	**0.49** (0.22–0.69)	**0.63** (0.40–0.78)	0.27 (−0.04–0.53)	**0.65** (0.43–0.80)	**0.81** (0.66–0.89)
**Fisher weighted mean correlation coefficient**			0.88	0.30	0.16	0.03	0.30	0.59	0.15	0.56	0.80

## 4. Discussion

Most locally-advanced solid tumors have heterogeneously-distributed hypoxic regions. This pattern makes quantitative assessment of PET data challenging, because uptake in a VOI represents the summation of tracer accumulation in different micro-regions. Therefore, data analysis with significantly smaller VOIs is more suitable for obtaining physiological parameters describing heterogeneous targets. Voxel-based kinetic analysis can be used to provide additional information of tracer behavior in small tissue sub-volumes, and a few kinetic studies have applied this approach to study tumor hypoxia and perfusion [37,38,40]. However, even though a voxel-based approach is better for the delineation of the heterogeneous distribution of PET tracers, the smaller size also results in a reduced signal-to-noise ratio. This can lead to inaccuracy in the estimation of parameters, and it is challenging to apply the correction algorithm for the partial volume effect [44,45,46]. Moreover, the larger number of voxels will result in extended computation time. Therefore, the choice of voxel size is a compromise between the signal-to-noise ratio and the ability to delineate the heterogeneous spatial distribution of hypoxic regions within a solid tumor. In this study, the voxel size of PET images was rescaled to 1.2 mm × 1.2 mm × 0.8 mm in order to reduce the effect of noise, but still provide the information of relatively small tumor sub-volumes. A similar voxel size has previously been used in a study comparing *K*_i_ obtained from dynamic ^18^F-FMISO PET with Po_2_ measurements [39]. Moreover, misalignment between CT and PET images could potentially lead to inclusion of tissue surrounding the tumor in the analysis. This can give rise to error generation in model parameter estimation. In order to minimize this effect, fiducial markers were used to ease image fusion.

TACs obtained from the left heart ventricle were used to generate input functions for pharmacokinetic analysis. Generally good fitting results were obtained for input function. This is important for model performance as the accuracy of the estimated kinetic parameters is influenced by the precision of the input function. As the signal-to-noise ratio in a TAC is closely related to the size of the VOI, it is difficult to compare different kinetic models based on voxel-wise fitting. Therefore, AIC was applied to compare two-tissue irreversible and reversible compartment models using TACs derived from larger VOIs covering whole tumor tissue. Based on this, the two-tissue irreversible compartment model was selected for the voxel-wise analysis and was used to calculate *k*_1_, *k*_3_ and *K*_i_. A strong correlation between *k*_1_ and early uptake of ^64^Cu-ATSM was found in this study, but this relationship was not found 30 min and 90 min pi (Table 2). As *k*_1_ can be considered as a transport parameter that is directly proportional with flow in the early stages of uptake, it is influenced by perfusion [37]. Studies of other hypoxic PET tracers have reported of similar relationships between early uptake and *k*_1_ [37,40,47]. In addition, ^64^Cu-ATSM uptake was compared to the tracer trapping parameter *k*_3_ and net influx rate *K*_i_. These kinetic parameters have previously been used to identify hypoxic tumor regions in dynamic PET studies using other PET tracers [38,39]. Most of the tumors in this study showed moderate to strong correlations between tumor uptake of ^64^Cu-ATSM 90 min pi and *k*_3_. *K*_i_ was less affected by noise than *k*_3_ and, in general, showed a strong correlation with late uptake.

The robustness of Cu-ATSM as a hypoxia marker has been questioned because of reports of temporal changes and tissue-specific differences in the uptake [29,48]. Moreover, *ex vivo* comparison has shown varying degrees of correlation between immunohistochemical markers of hypoxia and Cu-ATSM, and studies have found that factors other than hypoxia, such as pH and the redox status of the electron transport chain, can also affect the retention of the tracer [49,50,51,52]. In addition, a study has recently indicated that Cu-ATSM uptake is influenced by copper metabolism and that a fraction of the radiolabeled copper had dissociated from the ATSM complex shortly after administration in mice [53]. In clinical studies using Cu-ATSM, patients underwent PET imaging within the first 60 min pi, whereas pre-clinical studies have also included late time points, such as 18 h and 24 h pi [21,24,25,26,29,30,48]. No study has systematically evaluated the optimal time period between tracer administration and scanning, which is therefore currently unsettled. This is an important issue due to the potentially decreased tracer delivery to hypoxic tumor regions caused by limited perfusion. In addition, the influence of free ^64^Cu on the PET signal is likely to increase over time [53,54]. The relationship between ^64^Cu-ATSM accumulation 90 min pi and *k*_3_ and *K*_i_ indicates that the uptake at this time point is dominated by specific retention, and similar agreement of the late uptake (2–3 h pi) of nitroimidazole-based PET tracers to kinetic parameters used as indicators of hypoxia has been reported in other studies [36,40]. Therefore, our data suggests that 90 min pi may be a useful scan time for ^64^Cu-ATSM. However, the possible impact of ^64^Cu dissociation from the ATSM complex on the pharmacokinetic analysis is a limitation and has to be taken into account when interpreting the output from the pharmacokinetic analysis. Therefore, further studies are needed to evaluate the robustness of this relationship and compare parameters indicating a specific retention to other modalities, e.g., Po_2_ probe measurement.

Overall, dynamic PET data contain additional information on tracer behavior over time and kinetic, parameters that describe underlying physiological processes can be obtained. In hypoxia PET imaging, pharmacokinetic analysis can potentially be used to extract parameters, considered as surrogate markers of hypoxia, which accounts for differences in tracer delivery caused by variations in tumor perfusion. Even though this approach has shown promise, there is a need to evaluate these surrogate markers. Despite dynamic PET imaging in small animals being technically demanding due to the small size, mice and rats are attractive for this type of study, as they offer a high degree of flexibility and accessibility. In this study, voxel-wise kinetic analysis was performed in nude mice bearing subcutaneous tumors, and parameters of interest were successfully extracted from each voxel. The comparison of model parameters with ^64^Cu-ATSM uptake showed the feasibility to extract quantitative information that can be used for the validation of model output. Future studies will be focused on comparison between *k*_3_ and *K*_i_ against other image modalities and direct invasive tissue oxygen measurement.
